# Polymer Inclusion Membranes (PIM) for the Recovery of Potassium in the Presence of Competitive Cations

**DOI:** 10.3390/polym8030076

**Published:** 2016-03-15

**Authors:** Anna Casadellà, Olivier Schaetzle, Kitty Nijmeijer, Katja Loos

**Affiliations:** 1 Department of Polymer Chemistry, Zernike Institute for Advanced Materials, University of Groningen, Nijenborgh 4, 9747 AG Groningen, The Netherlands; Anna.Casadella@wetsus.nl; 2 Wetsus, European Centre of Excellence for Sustainable Water Technology, P.O. Box 1113, 8911 MA Leeuwarden, The Netherlands; Olivier.Schaetzle@wetsus.nl; 3 Membrane Science and Technology, MESA+ Institute for Nanotechnology, University of Twente, P.O. Box 217, 7500 AE Enschede, The Netherlands; d.c.nijmeijer@utwente.nl or D.C.Nijmeijer@tue.nl; 4 Membrane Materials & Processes, Department of Chemical Engineering & Chemistry Eindhoven University of Technology, Groene Loper 5, 5612 AE Eindhoven, The Netherlands

**Keywords:** polymer inclusion membrane, crown ether, potassium recovery, transport mechanism

## Abstract

Potassium is an important nutrient used in fertilizers but is not always naturally available  We investigated the properties of polymer inclusion membranes (PIM) regarding their selective recovery of K^+^ over competitive ions typically present in urine (Na^+^ and NH_4_^+^). The greatest flux was observed when the ratio of mass 2-nitrophenyl octyl ether (2-NPOE) used as plasticizer to cellulose triacetate (CTA) used as polymer was 0.25. The highest flux was achieved with a content of 24.8 wt % of dicyclohexan-18-crown-6 (DCH18C6) used as carrier, although the highest selectivity was observed with a content of 14.0 wt % of DCH18C6. We also studied whether the transport mechanism occurring in our system was based on co-transport of a counter-ion or ion exchange. Two different receiving phases (ultrapure water and 100 mM HCl) were tested. Results on transport mechanisms suggest that co-transport of cations and anions is taking place across our PIMs. The membrane deteriorated and lost its properties when the receiving phase was acidic; we suggested that this was due to hydrolysis of CTA. The greatest flux and selectivity were observed in ultrapure water as receiving phase.

## 1. Introduction

Nutrients used in fertilizers (e.g. potassium) are not always naturally available [1]. Urine is being more and more considered as a possible source of nutrients [2,3,4,5], so developing technologies to recover these nutrients is important as it can lead to a more circular chain of use and re-use of resources. The main cations present in urine (prior to urea hydrolysis) are: 49.0% sodium (Na^+^), 38.5% potassium (K^+^), 8.76% ammonium (${\text{NH}}_{4}^{+}$), 2.15% calcium (Ca^2+^) and 1.59% magnesium (Mg^2+^) [6]. Due to the high financial and energetic costs of ammonium production [7,8], studies based on its recovery from urine [9,10,11] will become more economically interesting in the future. Another cation interesting to recover from urine is K^+^ as it is not always an available nutrient [1] and it is widely used in fertilizers. Recovery of K^+^ closes a nutrient cycle based on the use of fertilizers, the human uptake and discharge of K^+^ (alimentation and excretion) and the further K^+^ reuse in fertilizers. When focusing on the main metal ions present in urine (K^+^, Na^+^ and NH_4_^+^), the challenge to selectively recover K^+^ is that K^+^, Na^+^ and NH_4_^+^ have same charge (+1) and very similar hydrated radii [12] which renders separation by size exclusion unsuitable. Moreover, K^+^ and NH_4_^+^ present a very similar diffusion coefficient [13] which makes diffusivity an unsuitable tool for the separation process (Table 1).

In the last years, several studies have shown the possibility of recovering cations using solvent extraction as well as the transport through liquid membranes (LMs) [14,15,16,17]. LMs can be found in different forms *i.e.*, bulk (BLMs), emulsion (ELMs) and supported (SLMs). However, they present poor stability and flux. Consequently membrane technology evolved towards polymer inclusion membranes (PIMs) [18,19]. PIMs can separate and recover small organic molecules from an aqueous mixture as well as transport metal ions with high selectivity and flux. For example, Schow *et al.* [20] showed that the flux through a PIM was three orders of magnitude higher than for a SLM under the same conditions. PIMs are composed of polymer, plasticizer and carrier [18]. Polymers provide mechanical strength to the membrane. The most common polymers used for preparing PIMs are cellulose triacetate (CTA) and poly(vinyl chloride) (PVC) due to their high solubility in organic solvents. Plasticizers (e.g., 2-nitrophenyl octyl ether, 2-NPOE) are generally used to lubricate the segment motions of polymers (and therefore increase the ion flux across the membrane) and to provide flexibility. Carriers are found in different types: basic (e.g., quaternary amines [21]), acidic and chelating (e.g., sulfonic acids [22]), neutral (e.g., phosphoric acid esters [23]), macrocyclic and macromolecular (e.g., imidazole azothiacrown ethers [24]). For the transport of alkali metals (e.g., K^+^), macrocyclic and macromolecular crown ethers are commonly used as carriers [25]. They have a specific host-guest complexation behavior which allows the transport of the target ion across the PIM.

**Figure 1:**
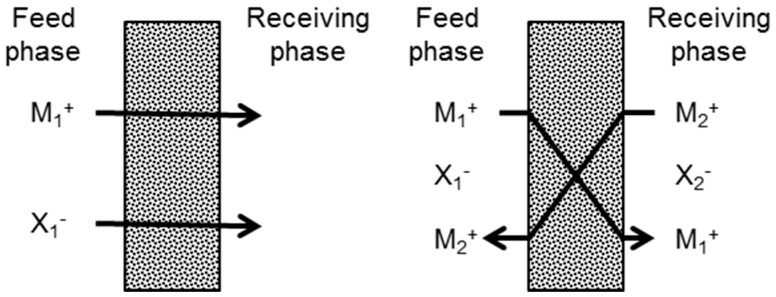
Scheme of the transport across a PIM for a cation (M_n_^+^) and an anion (X_n_^−^): co-transport of a counter-ion (**left**); and ion-exchange (**right**).

PIMs for the separation of K^+^ have already been developed. For instance, Schow *et al.* [20] used dicyclohexan-18-crown-6 (DCH18C6) for the recovery of K^+^ from rubidium (Rb^+^) and sodium (Na^+^). Heng *et al.* [26] used a natural ionophore (valinomycin) to assess the selectivity for K^+^ of a new class of polymers based on methacrylate-acrylate to be further used in ion-selective electrodes (ISE) [27]. Thunhorst *et al.* [25] cross-linked benzo-18-crown-6 and acrylate to achieve a more robust membrane and assess its selectivity for K^+^ over Na^+^ and Rb^+^. Each of these studies used ultrapure water as a stripping phase in the receiving compartment when assessing the transport properties of the membranes. Therefore, in order to preserve electroneutrality, the target ion is transported together with its counter-ion (co-transport, Figure 1 (left)) across the membrane to the receiving phase. However, other studies used an acidic solution in the receiving compartment as a source of counter-ions for the diffused target ion [24,28,29,30,31,32] (ion-exchange, Figure 1 (right)). Benosmane *et al.* [33] studied the parameters for the best transport of metal ions through a PIM. They showed that diffusion transport of Pb^2+^ (calixarene as carrier) was dependent on the pH of the receiving phase. Transport of Pb^2+^ increased up until pH 5.5-6 were flux decreased due to membrane deterioration.

In order to design a proper recovery technology for the separation of nutrients from urine, we studied the membrane performance in terms of selectivity and transport of competitive cations that not only have the same charge but also have similar hydrated radii and a similar diffusion coefficient. We evaluated an optimal composition of PIM to selectively recover K^+^ from equimolar solutions of the main cations in urine (K^+^, Na^+^, NH_4_^+^). We studied the influence on transport and selectivity of each of the components of the membrane: cellulose triacetate (CTA), plasticizer (2-nitrophenyloctyl ether, 2-NPOE) and a macromolecular carrier (dicyclohexan-18-crown-6, DCH18C6) that has already been reported as suitable for K^+^ selective PIMs [20]. Furthermore, we suggest a transport mechanism for our optimized PIM based on the influence of different receiving solutions (ultrapure water and acid) on transport and selectivity to give more insight into the optimal conditions of the process for further scale-up. 

## 2. Materials and Methods

### 2.1. Chemicals

Potassium nitrate (KNO_3_), sodium nitrate (NaNO_3_), ammonium nitrate (NH_4_NO_3_), cellulose triacetate (CTA), 2-nitrophenyl octyl ether (2-NPOE), dichloromethane (DCM), hydrochloric acid (HCl, 37%), and dicyclohexano-18-crown-6 (DCH18C6) were purchased from Sigma Aldrich (Zwijndrecht, The Netherlands). All chemicals (highest purity grade) were used without further purification. Aqueous solutions were prepared using ultrapure water obtained by a Millipore purification unit (Millipore B.V., Amsterdam, The Netherlands). 

### 2.2. Membrane Preparation

Membranes were prepared following the procedure reported by Schow *et al.* [20] and Sugiura *et al.* [34]. Solutions in DCM of different proportions of CTA (25.0 g·L^−1^), DCH18C6 (18.6 g·L^−1^, 50.0 mM) and 2-NPOE (without further dilution), were used to produce membranes with a total weight of 0.3 g excluding the solvent. The different compositions in weight percentage used are shown in Table 2. The corresponding mixture of each membrane was placed in a 9 cm diameter flat bottom glass Petri-dish. The dish was put in a flat box under nitrogen atmosphere overnight to allow the solvent to evaporate slowly and have little contact with air humidity, thus to avoid formation of pores. Then, membranes were peeled off the dish by adding a few droplets of ultrapure water. The resulting membranes had a thickness of 30 ± 6.0 µm which was measured with a thickness gauge.

### 2.3. Membrane Characterization

#### 2.3.1. Transport Experiments

**Figure 2:**
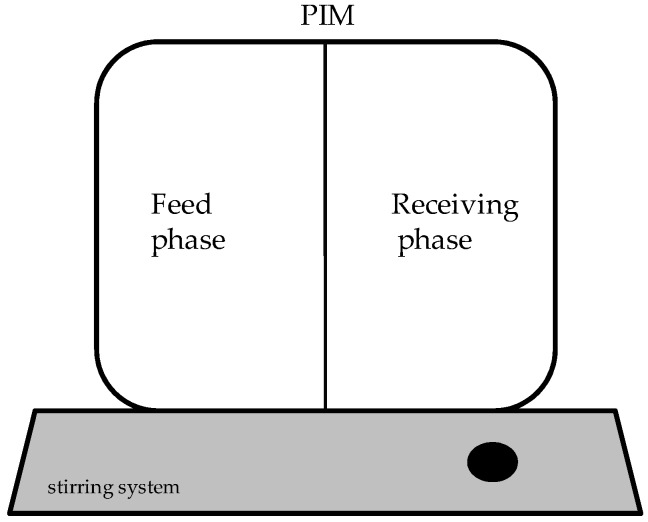
Scheme of the diffusion cell.

To assess the transport of ions, synthesized PIMs were clamped between the two compartments of a diffusion cell on a Teflon ring-shaped support (Figure 2). The diffusion cell was made of poly(methyl) methacrylate (PMMA) (STT, Leeuwarden, The Netherlands). PIMs under study had a working area of 7.07 cm^2^. For reference, the side of the membrane exposed to the nitrogen atmosphere (upper side of the membrane in the Petri-dish) was placed facing the receiving compartment. Each of the compartments of the diffusion cell had a capacity of 100 mL and phases were homogenized by stirring at a speed of 500 rpm with magnetic bars. The feed compartment was filled with a total concentration of 0.1 M aqueous solution containing the nitrate salts (0.03 M of each salt when mixed), and the receiving compartment was filled with either ultrapure water or 0.1 M HCl, depending on which transport mechanism is studied. All measurements were carried out at 25 ± 2 °C controlled by a temperature sensor (QM701T, QIS, ProSense B.V., Oosterhout, The Netherlands).

Samples of 1 mL were taken from each compartment at different time intervals. The volume difference was compensated for by adding 1 mL of ultrapure water in the corresponding compartment after every sample. Because of the addition of ultrapure water, a dilution is induced in the phases so it was taken into account during calculations.

Flux J_i_ (mmol·cm^−2^·h^−1^) across the membranes for each of the ions was calculated as:

$$J_{i}=\frac{V}{A}\frac{{dC}_{i}}{dt}$$

where *V* (L) is the volume of the compartment, *A* (cm^2^) is the membrane working area and $\frac{{dC}_{i}}{dt}$ (mmol_i_·L^−1^) is the concentration change in time in the receiving phase.

The relative selectivity between two different ions ($\alpha_{i,j}$) (−) was calculated as:

$$\alpha_{i,j}=\frac{J_{i}}{J_{j}}\frac{{\Delta C}_{j}}{{\Delta C}_{i}}$$

where Δ*C*_i,j_ is the concentration difference of each of the ions (*i* and *j*) between the two compartments after 50 h.

#### 2.3.2. Analyses

To determine the mass balance in both compartments, the concentration of K^+^, Na^+^, NH_4_^+^, Cl^−^ and NO_3_^−^ was determined by ion-chromatography (IC, Metrohm Compact, for cations IC 761 and anions IC Pro 881, Schiedam, The Netherlands). Although in this article only the data of the receiving phase is shown, the mass balance fits in all experiments. Proton (H^+^) transport was measured via pH variations using a pH-meter (Metrohm 827 pH lab, Schiedam, The Netherlands,). Release of 2-NPOE and DCH18C6 was analyzed by LC-MS (Agilent 1200 series, column: G1316B-6410 Triple Quad, Agilent Technologies, Amstelveen, The Netherlands) using as mobile phase a solution (25:75) of formic acid-ammonia buffer at pH 8.75 and acetonitrile with 0.1 *v*/*v* % formic acid.

## 3. Results and Discussion

### 3.1. Effect of CTA and 2-NPOE on K^+^ Transport

First, diffusion properties of the synthesized membranes (Table 2) were tested to assess their K^+^ flux. Unless stated otherwise, the receiving solution was ultrapure water.

#### 3.1.1. Effect of CTA

Polymers composed of cellulosic units such as CTA form membranes with two types of regions: crystalline and amorphous. In crystalline regions, where polymer chains are distributed in a systematic fashion, CTA forms hydrogen bonds and van der Waals forces, therefore CTA holds together and spaces between polymer chains are definite. In amorphous regions, where polymer chains do not follow any systematic order, spaces between polymer chains are greater. Therefore, water molecules in CTA membranes accumulate in the amorphous regions (greater spaces), resulting in hydrogen bonds between CTA and water so voids in the structure are reduced. Transport of ions is then dependent on the hydrogen bonds in the CTA membrane. Ions can transport through the voids hydrogen bonds are not present or combine with the hydrogen bonding [35] regarding their hydrated radii [36]. To evaluate the behavior of K^+^ in our PIM a control membrane (PIM-1) in absence of carrier and plasticizer was assessed to study the influence of the polymer (CTA) on the flux of K^+^. Results in Table 3 show the flux of K^+^ (10^−3^·mmol·cm^−2^·h^−1^) in the receiving phase after 50 h. CTA allows a flux of 5.70 × 10^−3^ mmol K^+^ cm^−2^·h^−1^ across PIM-1. Therefore, CTA is not completely impermeable to K^+^ which suggests that PIM-1 presents some voids for K^+^ to be transported, as K^+^ does not have the capacity to create hydrogen bonds.

#### 3.1.2. Effect of CTA and 2-NPOE

To overcome the rigidity based on van der Waals forces between polymer chains and water molecules formed in the CTA membrane structure, plasticizers are added. Plasticizers are known to reduce van der Waals forces and hydrogen bonds between polymer molecules and water molecules [37]. Therefore, PIMs become more flexible and the ion flux is increased as the presence of voids is increased as well [18]. Because the role of the plasticizer is important for PIMs, we studied its effect on the flux of K^+^. Table 3 presents the effect of the content of 2-NPOE (plasticizer) on the flux of K^+^ across synthesized PIMs without the presence of the carrier (PIM-2–5). As the content of 2-NPOE increased, the content of CTA decreased as membrane weight was set to be constant (0.3 g). Its measured thickness (30 µm) was as well constant.

Different compositions of 2-NPOE and CTA lead to a change in the flux of K^+^ in 50 h. The presence of 20 wt % of 2-NPOE (PIM-2) increased the flux of K^+^ (7.02 × 10^−3^·mmol K^+^·cm^−2^·h^−1^) almost 10-fold compared with PIM-1 (0.57 × 10^−3^ mmol K^+^·cm^−2^·h^−1^) which had no 2-NPOE. However, a higher presence of 2-NPOE such as 40 and 60 wt % (PIM-3 and PIM-4) led to a decrease of the flux of K^+^ being 4.38 × 10^−3^ mmol K^+^·cm^−2^·h^−1^ for PIM-3 and 1.90 × 10^−3^ mmol K^+^·cm^−2^·h^−1^ for PIM-4. The decrease in the flux of K^+^ is attributed to the hydrophobic nature of 2-NPOE. The higher the content of 2-NPOE in the PIM structure, the more hydrophobic the membrane becomes and the lower the affinity is of K^+^ towards the PIM. Therefore a ratio of 2-NPOE *versus* CTA of 0.25 was found to be the optimum for our study. PIM-5 consisted of 20 wt % CTA and 80 wt % 2-NPOE but was not tested because its lack of mechanical strength.

### 3.2. Effect of the PIM Composition on K^+^, Na^+^ and NH_4_^+^ Flux and Selectivity

#### Effect of DCH18C6

To assess the influence of DCH18C6, membranes with a 2-NPOE *versus* CTA ratio of 0.25 with different DCH18C6 contents were tested for flux and selectivity using an equimolar mixture of K^+^, Na^+^ and NH_4_^+^ in the feed phase.

- Effect on the Cation Flux

**Figure 3:**
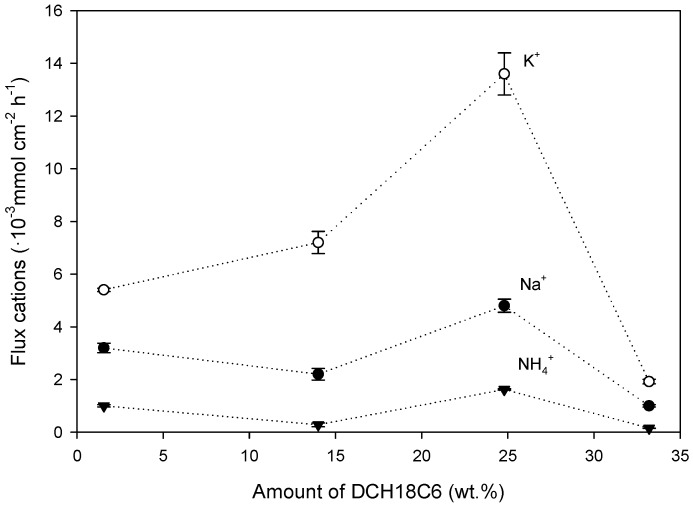
Effect of DCH18C6 content on the flux of the optimized PIM after 50 h. Results correspond to PIM-6 (1.56 wt %), -7 (14.0 wt %), -8 (24.8 wt %) and -9 (33.2 wt %).

**Figure 4:**
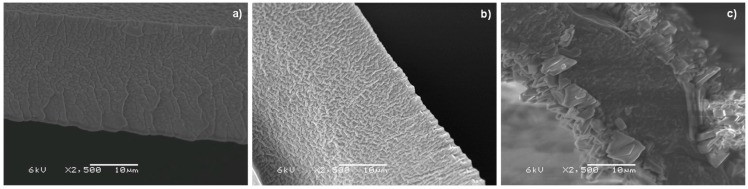
SEM images (magnification: 2500×) of cross-section PIMs with contents of DCH18C6 of 14.0 wt % (**a**); 24.8 wt % (**b**); and 33.2 wt % (**c**).

As shown in Figure 3, the flux for K^+^ of PIMs containing DCH18C6 increased by 10-fold compared to PIM-1 containing only CTA and by up to 2-fold compared to PIM-2 containing CTA and 2-NPOE. Flux across the membrane also depends on the distance between each of the carriers. If this distance is small enough, it is more feasible for cations to be transported through the percolation path [38]. For any content of DCH18C6, the flux of K^+^ was greater than for NH_4_^+^ and Na^+^. PIM selectivity is later discussed. Regarding the overall flux, PIM-7 with a content of DCH18C6 of 14.0 wt % presented a lower flux of cations across the membrane than PIM-8 that contained 24.8 wt % of DCH18C6. However, 24.8 wt % is a critical content of DCH18C6 above which the flux decreased drastically (*i.e.*, from 13.6 × 10^−3^ mmol K^+^·cm^−2^·h^−1^ down to 2.0 × 10^−3^ mmol K^+^·cm^−2^·h^−1^). To provide an explanation, SEM images were taken to evaluate the morphological properties of three membranes: PIM-7 with a lower content of DCH18C6 (14.0 wt %) (Figure 4a) than the critical content, PIM-8 with the critical content of DCH18C6 (24.8 wt %) (Figure 4b) and PIM-9 with a higher content (33.2 wt %) (Figure 4c). Contents of DCH18C6 higher than the critical content presented a precipitate on the surface of the membrane. This indicates that DCH18C6 was not soluble anymore and formed layers on the polymeric matrix, which leads towards a drastic decrease of cation flux as the precipitated DCH18C6 did not promote the mobility of ions across the membrane (jumping mechanism is blocked). This phenomenon was also experienced by Gherrou *et al.* [38] who used dicyclobenzo-18-crown-6 (DB18C6) to recover copper.

- Effect on selectivity

Selectivity (α_i,j_) was assessed comparing the content of K^+^ transported in comparison with the content of Na^+^ and NH_4_^+^ (competitive cations) transported after 50 h. As shown in Table 4, the optimum content of DCH18C6 to achieve the highest selectivity of K^+^ over Na^+^ and NH_4_^+^ (PIM-7, 14.0 wt %) did not correspond with the content needed for the highest flux of cations (PIM-8, 24.8 wt %). Our hypothesis is that an increase of the content of DCH18C6 present in the membrane provides more available sides for cations to be transported, so flux for PIM-8 is greater than PIM-7. However, selectivity also presents an optimum. The same behavior for the selectivity of K^+^ over Na^+^ is experienced for PIM-9 although selectivity of K^+^ over NH_4_^+^ increases. It would suggest that NH_4_^+^ would be retained in the feed compartment, possibly due to hydrogen bond formation with the precipitated DCH18C6.

### 3.3. Transport Mechanism

Depending on the actual transport mechanism of cations and anions across the membrane, the solution in the receiving phase plays an important role. Two possible mechanisms are distinguished: co-transport and ion exchange [18]. To assess which transport mechanism takes place in our K^+^ selective PIMs we tested two receiving phases, ultrapure water (co-transport) and 100 mM HCl (ion exchange), for the membrane that showed the best performance (PIM-8). Furthermore, we compared the effect of the two receiving phases on PIM-8 in terms of selectivity and stability. The feed phase contained solutions of 100 mM of the corresponding salt.

#### 3.3.1. Effect of the Receiving Phase on the Transport Mechanism

**Figure 5:**
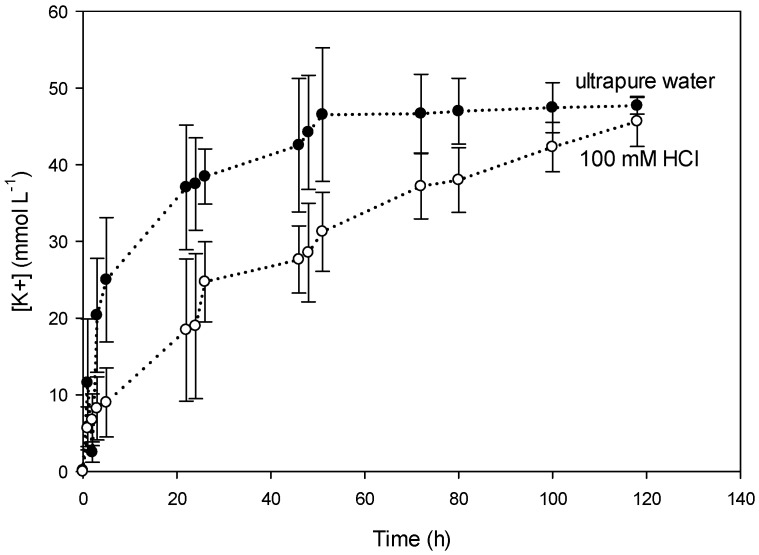
Evolution using PIM-8 of [K^+^] in the receiving compartment in two different receiving phases: ultrapure water and 100 mM HCl.

**Figure 6:**
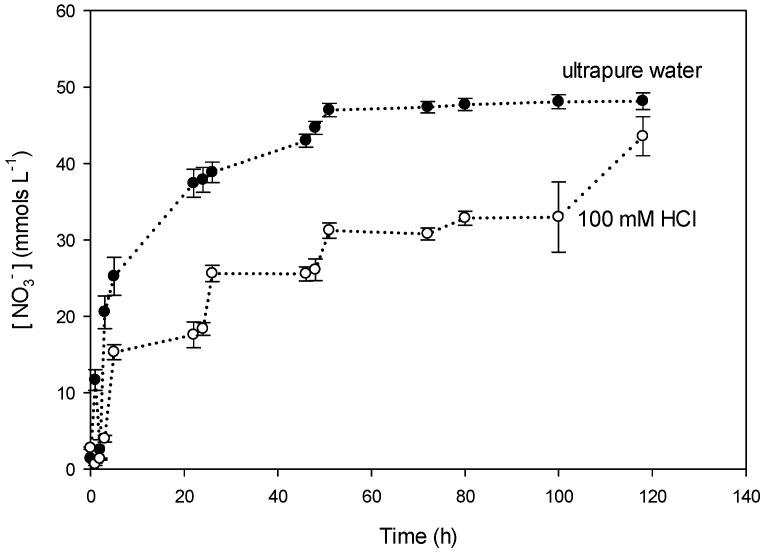
Evolution using PIM-8 of [NO_3_^−^] in the receiving compartment in two different receiving phases: ultrapure water and 100 mM HCl.

Figure 5 shows the concentration of K^+^ in the receiving compartment in time using two different receiving phases: ultrapure water and 100 mM HCl. For the receiving phase containing water, equilibrium (50 mM K^+^) is reached after 50 h and for the acidic phase equilibrium is reached after 120 h. This suggests that the presence of acid in the receiving solution slows down the diffusion of K^+^ across the PIM. Moreover, Figure 6 shows the diffusion of NO_3_^−^ for the same types of receiving solutions. Equilibrium of NO_3_^−^ was reached at the same time as K^+^ regarding the receiving solution: 50 h for ultrapure water and 120 h for 100 mM HCl. At equilibrium the concentration of K^+^ and NO_3_^−^ was around 50 mM. The difference in time in reaching equilibrium regarding the receiving phase could be due to the stability of CTA in the PIM (hydrolysis) as described later in this paper in the study on the membrane stability.

**Figure 7:**
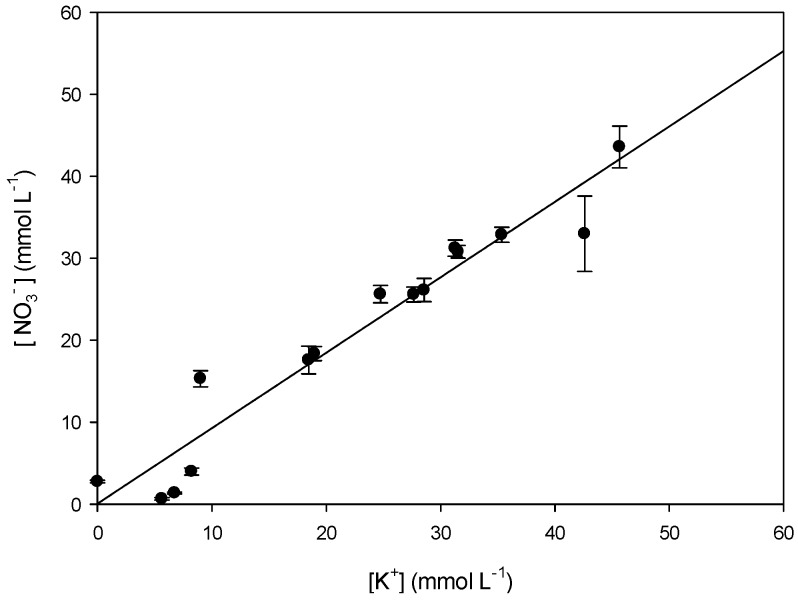
Comparison of the transported [K^+^] and [NO_3_^−^] in the receiving phase of ultrapure water using PIM-8.

**Figure 8:**
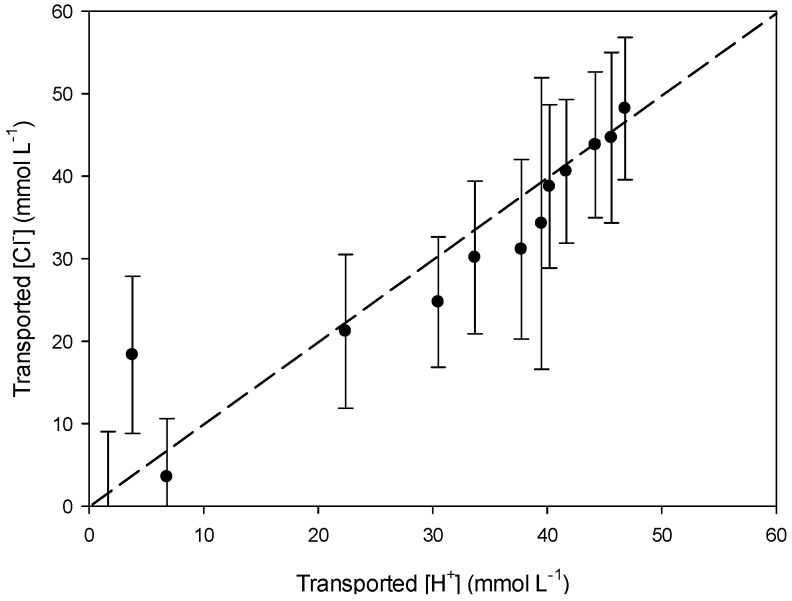
Comparison of the transported [H^+^] and [Cl^−^] in the feed phase using PIM-8.

**Figure 9:**
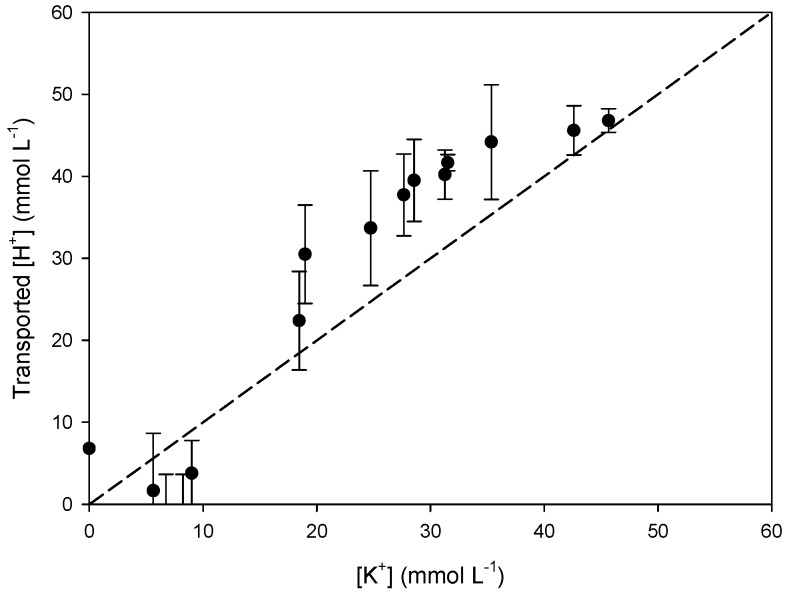
Comparison of the transported [H^+^] into the feed phase and [K^+^] in the receiving phase using PIM-8.

**Figure 10:**
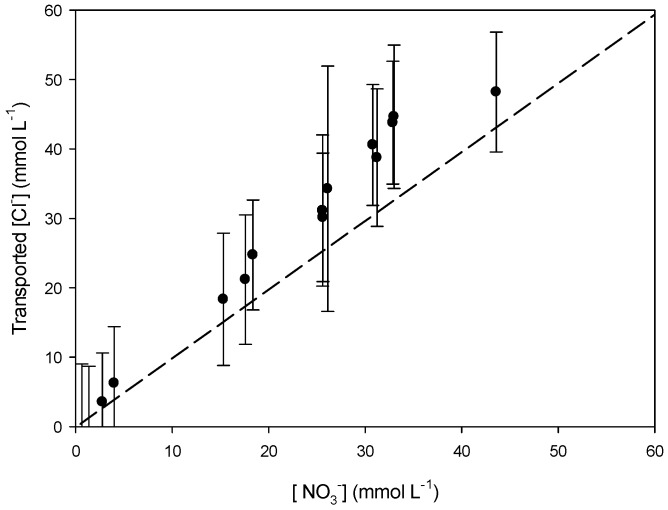
Comparison of the transported [Cl^−^] into the feed phase and [NO_3_^−^] into the receiving phase using PIM-8.

To study which transport mechanism is applicable in our system, we studied the relation between K^+^ and NO_3_^−^ and H^+^ and Cl^−^ transport in/from the receiving compartment. The slopes of the linear regression curves (*y* = a*x* + b) of Figure 7 (a = 0.92) and Figure 8 (a = 1.05) are very close to 1. This suggests that K^+^ and NO_3_^−^ (Figure 7) are transported as a pair across PIM-8 from the feed to the receiving compartment and that H^+^ and Cl^−^ are as well transported together to the feed compartment (Figure 8), so co-transport could be the transport mechanism occurring in the system (Table 5):

To confirm this hypothesis, the relation between K^+^ and H^+^ (Figure 9) and Cl^−^ and NO_3_^−^ (Figure 10) was determined as well and a parity plot was used as reference (dashed line). The slopes of both regression curves are above 1 (a = 1.24) meaning that there is no coupled relation (co-transport) between the corresponding cations and anions. Consequently, co-transport seems to be the main transport mechanism in our system.

#### 3.3.2. Effect of the Receiving Phase on Selectivity

Previously presented results show that an acidic receiving phase is not optimal for high flux transport of the target cation. However, another parameter to compare the performance of membranes is selectivity (Table 6). Membranes with the same composition as the previously optimized PIM (PIM-8) were tested for their selectivity using ultrapure water or 100 mM HCl in the receiving compartment. Whereas equilibrium was reached after 50 h when the receiving solution was water, equilibrium is reached after 120 h when the receiving solution is 100 mM HCl. Table 5 shows the flux of K^+^, Na^+^ and NH_4_^+^ after 50 h for both receiving phases and after 120 h for the acidic receiving phase. Again, the flux of K^+^ is higher (6.8 × 10^−3^ mmol·cm^−2^·h^−1^) in case of water as receiving phase than in case of acid (3.8 × 10^−3^ mmol·cm^−2^·h^−1^). However, fluxes of Na^+^ and NH_4_^+^ are very similar in both types of receiving phases which suggests that the percolation path of DCH18C6 is only affected for K^+^. Selectivity in ultrapure water is also greater than in acid. Average selectivity of K^+^ over Na^+^ is 21.4 in ultrapure water and 11.6 in acid. Average selectivity of K^+^ over NH_4_^+^ is 86.0 in ultrapure water and 38.3 in acid. However, in acid, at 120 h (equilibrium) selectivity is reduced from 11.6 to 5.30 for K^+^ over Na^+^ and from 38.3 to 14.8 for K^+^ over NH_4_^+^. The difference in flux and the reduction of selectivity, suggests that ultrapure water is the best to use in this case as receiving phase. Moreover, these results also suggest that the membrane is losing its properties over time in contact with acidic conditions. Therefore, we investigated the membrane stability in contact with the receiving phase.

#### 3.3.3. Effect of the Receiving Phase on Membrane Stability

**Figure 11:**
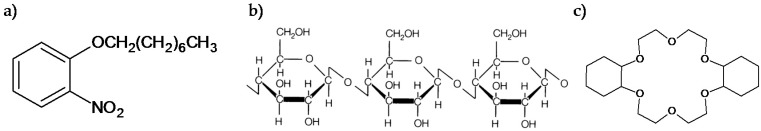
Chemical structure of 2-NPOE (**a**); CTA (**b**); and DCH18C6 (**c**).

Any possible 2-NPOE and DCH18C6 release from the membrane was studied to assess composition stability of the PIM. Samples were taken from both compartments (feed and receiving) and three types of receiving phases: ultrapure water and acidic (10 mM HCl and 100 mM HCl). The release in feed compartment and receiving for all receiving phases (ultrapure water and HCl) was equivalent, so we show the measurements in the different receiving phases. Measurements of the release of 2-NPOE showed that the presence of 2-NPOE in both compartments and types of phases was not detectable. The concentration of 2-NPOE was below 6 µg**·**L^−1^ (limit of detection of LC-MS). This suggests that the hydrophobic nature of 2-NPOE (Figure 11a) prevents its detectable release from the PIM as it does not present affinity for aqueous solutions.

**Figure 12:**
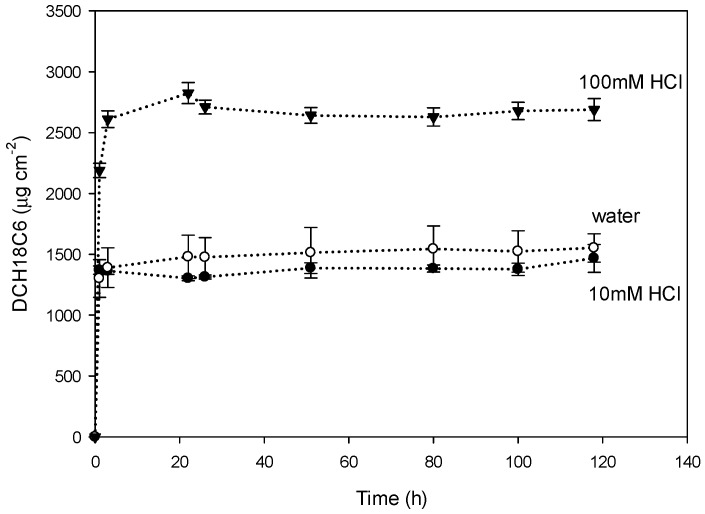
Release of DCH18C6 from PIM in three receiving phases: water, 10 mM HCl and 100 mM HCl.

Figure 12 shows the content of DCH18C6 (chemical structure in Figure 11c) released per area of membrane in time for the three types of receiving phases: water, 10 mM HCl and 100 mM HCl. For each of the cases, during the first 3 h there was a release of DCH18C6 into the receiving phase. Later, DCH18C6 was not released anymore as its content remained stable until the experiment was finished. There were differences in the content of DCH18C6 released depending on the receiving phases. Receiving phases containing water or 10 mM HCl presented similar behavior; the content of DCH18C6 released was 1500 µg per cm^2^ of PIM for both phases, whereas for the receiving phase containing 100 mM HCl, the content of DCH18C6 released was 2700 µg per cm^2^ of PIM. Therefore, more DCH18C6 was released when the receiving phase was 100 mM HCl. This can be explained because under acidic conditions CTA is prone to hydrolysis due to the presence of esters in its chemical structure (Figure 11b). Hydrophobic non-polar carriers (*i.e.*, DCH18C6) become incompatible in such environment [39,40] because the loss of the methyl groups by hydrolysis leads to a less hydrophobic membrane interface. Therefore, DCH18C6 is not soluble anymore and it releases from the membrane matrix. The release of DCH18C6 in the acidic phase (100 mM HCl) corresponds to 0.21% of the total content of DCH18C6 in the membrane and for the water phase (and 10 mM HCl) the release corresponds to 0.12%. The content of DCH18C6 released for either receiving phases we consider it not to be significant in altering the flux and selectivity. However, the hydrolysis of CTA by acidic conditions, as Gardner *et al.* [39] also showed, could have a significant effect on the transport and selectivity of CTA-based PIMs.

## 4. Conclusions

We prepared a series of PIMs with different compositions of CTA, 2-NPOE and DCH18C6. Each of the components has been assessed for its effect on the flux of K^+^ and its competitive cations (Na^+^ and NH_4_^+^). The greatest flux was observed when the ratio of 2-NPOE *versus* CTA was 0.25 and the content of DCH18C6 was 24.8 wt %, although highest selectivity was obtained with a content of 14.0 wt % of DCH18C6. Also, two different receiving phases (ultrapure water and 100 mM HCl) were used to study the transport mechanism of the PIMs, their selectivity and their stability. Results suggest that co-transport of the counter-ion is the governing transport mechanism across our PIMs. We hypothesize that due to the hydrolysis of CTA in acidic conditions the membrane deteriorated and lost its properties. Thus, highest flux and selectivity were observed when the receiving phase was ultrapure water.

## Figures and Tables

**Table 1:** Comparison of hydrated radii and diffusion coefficient (in water) of main metal ions present in urine [12,13].

Cation	Hydrated radii (Å)	Diffusion coefficient (10^−5^·cm^2^·s^−1^)
K^+^	3.31	1.96
Na^+^	3.58	1.33
NH_4_^+^	3.31	1.96

**Table 2:** Weight percentage (wt %) of DCH18C6, CTA and 2-NPOE for each of the prepared membranes.

Assigned name	DCH18C6 (wt %)	CTA (wt %)	2-NPOE (wt %)
PIM-1	0.0	100	0.0
PIM-2	0.0	80.0	20.0
PIM-3	0.0	60.0	40.0
PIM-4	0.0	40.0	60.0
PIM-5	0.0	20.0	80.0
PIM-6	1.56	78.7	19.7
PIM-7	14.0	68.8	17.2
PIM-8	24.8	60.2	15.0
PIM-9	33.2	53.4	13.4

**Table 3:** Effect of the content of plasticizer in PIM. Fluxes monitored in the receiving compartment.

Assigned name	Ratio 2-NPOE *vs.* CTA	Content CTA (wt %)	Content 2-NPOE (wt %)	Content CTA (10^−2^·g·cm^−2^)	Content 2-NPOE (10^−2^·g·cm^−2^)	*J*_K_ (10^−3^ mmol·cm^−2^·h^−1^)
PIM-1	0.0	100	0.0	4.70	0.0	0.57 ± 0.04
PIM-2	0.25	80.0	20.0	3.76	0.94	7.02 ± 0.32
PIM-3	0.67	60.0	40.0	2.82	1.88	4.38 ± 0.24
PIM-4	1.50	40.0	60.0	1.88	2.88	1.90 ± 0.08

**Table 4:** Selectivity of K^+^ over Na^+^ and NH_4_^+^ regarding the content of DCH18C6 in the PIM.

Assigned name	DCH18C6 (wt %)	Content of DCH181C6 (10^−3^g cm^−2^)	α_Na,K_ (-)	α_NH4,K_ (-)
PIM-6	1.56	0.07	5.35	50.1
PIM-7	14.0	0.66	27.9	295
PIM-8	24.8	1.18	21.4	86.0
PIM-9	33.2	1.55	7.02	133

**Table 5:** Co-transport mechanism of KNO_3_ in PIM containing carrier.

Feed phase	PIM bulk	Receiving phase
K^+^ NO_3_^−^ →	K^+^[DCH18C6] ... NO_3_^−^ →	K^+^ NO_3_^−^
H^+^ Cl^−^	← H^+^ Cl^−^	← H^+^ Cl^−^

**Table 6:** Selectivity and flux of K^+^, Na^+^, NH_4_^+^ after 50 and 120 h. Receiving solution was ultrapure water or 100 mM HCl.

Time (h)	Receiving phase	*J* (10^−3^ mmol·cm^−2^·h^−1^)	*J*_Na_ (10^−3^ mmol·cm^−2^·h^−1^)	*J*_NH4_ (10^−3^ mmol·cm^−2^·h^−1^)	α_Na,K_ (−)	α_NH4,K_ (−)
50	water	6.8 ± 0.4	2.4 ± 1.4	0.8 ± 0.1	21.4	86.0
	HCl	3.8 ± 0.6	2.0 ± 0.3	0.7 ± 0.1	11.6	38.3
120	water	N/A	N/A	N/A	N/A	N/A
	HCl	6.3 ± 0.9	5.8 ± 0.7	3.8 ± 0.7	5.30	14.8

N/A: not applicable.

## Abbreviations

The following abbreviations are used in this manuscript:

A	area
BLM	bulk liquid membrane
CTA	cellulose triacetate
DCM	dichloromethane
DCH18C6	dicyclohexan-18-crown-6
ELM	emulsion liquid membrane
ISE	ion-selective electrode
Ji	flux ion i
LC-MS	liquid chromatography—mass spectrometry
Mn^+^	cation
PIM	polymer inclusion membrane
PMMA	poly (methyl) acrylate
PVC	polyvinyl chloride
SEM	scanning electron microscope
SLM	supported liquid membrane
V	volume
Xn-	anion
2-NPOE	2-nitrophenyl octyl ether
αi,j	relative selectivity
